# A guided approach to diagnose severe muscle weakness in the intensive care unit

**DOI:** 10.5935/0103-507X.20150036

**Published:** 2015

**Authors:** Nicola Latronico, Rik Gosselink

**Affiliations:** 1 Department of Anesthesia, Critical Care Medicine and Emergency, Spedali Civili University Hospital - Brescia, Italy.; 2 Department of Medical and Surgical Specialties, Radiological Sciences and Public Health, University of Brescia - Brescia, Italy.; 3 Department of Rehabilitation Sciences, Katholieke Universiteit Leuven - Leuven, Belgium.

Intensive care unit (ICU) acquired muscle weakness (ICUAW) is a clinically detected condition characterized by diffuse, symmetric weakness involving the limbs and respiratory muscles.^(1)^ Patients have different degrees of limb muscle weakness and are dependent on a ventilator, while the facial muscles are spared. Diagnosis of ICUAW requires that no plausible etiology other than critical illness be identified, and thus, other causes of acute muscle weakness are excluded. One major diagnostic criterion is that ICUAW is detected after the onset of critical illness; therefore, it is important to differentiate ICUAW from Guillain-Barrè syndrome or other acute neuromuscular disorders that may cause respiratory failure and ICU admission (Figure 1).^(1)^ The use of neuromuscular blocking agents for long periods of time, the use of some antibiotics and electrolyte abnormalities, such as hypermagnesemia, hypokalemia, hypercalcemia, and hypophosphatemia, and prolonged immobilization are common in the ICU and should be appropriately treated before a diagnosis of ICUAW is posed.^(2)^

**Figure 1:**
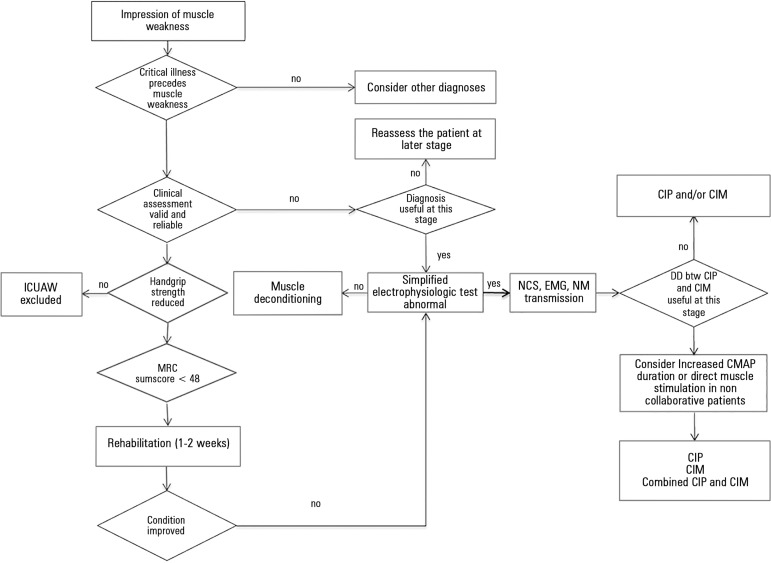
Diagnostic algorithm for intensive care unit acquired muscle weakness (ICUAW). Modified from: Latronico N, Bolton CF. Critical illness polyneuropathy and myopathy: a major cause of muscle weakness and paralysis. Lancet Neurol. 2011;10(10):931-41.^(1)^ Cut-off handgrip strength values were below 7kg for female and below 11Kg for males. DD - differential diagnosis; NCS - nerve conduction study; EMG - electromyography; NM - neuromuscular; CIP - critical illness polyneuropathy; CIM - critical illness myopathy; MRC - Medical Research Council; CMAP - compound muscle action potential.

A diagnosis of ICUAW is achieved by manually testing the muscle strength using the Medical Research Council (MRC) scale or by measuring handgrip strength using a dynamometer.

MRC muscle strength is assessed in 12 muscle groups (Figure 2): a summed score below 48/60 designates ICUAW or significant weakness, and an MRC score below 36/48 indicates severe weakness.^(3)^ Recently, a simplified version of the scale with only four categories and improved clinimetric properties was proposed (Figure 2).^(4)^ To date, this version has been validated in a small cohort of 60 critically ill patients with excellent inter-rater reliability and high sensitivity and specificity in diagnosing ICUAW compared to complete full MRC.^(5)^

**Figure 2:**
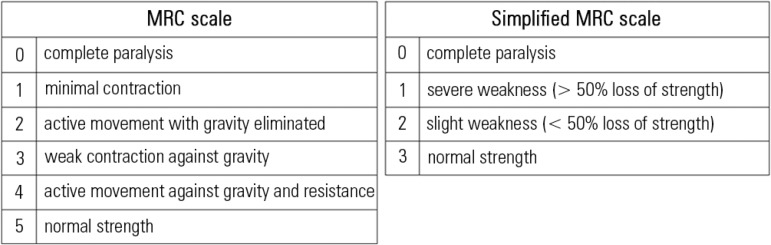
Original and simplified Medical Research Council (MRC) scales. Both scales are bilaterally applied to six muscle groups of the upper and lower limbs in order to obtain a summed score ranging from 0 to 60 for the classic MRC scale and from 0 to 36 for the simplified version: (1) abduction of the arm; (2) flexion of the forearm; (3) extension of the wrist; (4) flexion of the leg or hip flexion; (5) extension of the knee; and (6) dorsal flexion of the foot.

Handgrip dynamometry measures isometric muscle strength and can be used as a quick diagnostic test. Cut-off scores of less than 11kg (IQR 10 - 40) in males and less than 7kg (IQR 0 - 7.3) in females are considered to be indicative of ICUAW (Figure 1).^(5)^ Both MRC and handgrip dynamometry are volitional tests and require the patients to be alert, cooperative, and motivated. Sedation, delirium and coma often interfere with the early evaluation of muscle strength in the ICU. However, voluntary muscle strength using the MRC sum score or handgrip dynamometry can be reliably assessed if adequate clinical experience is gained with manual muscle testing in ICU patients and strict guidelines and the use of standardized test procedures and positions are followed to accurately select patients.^(6)^

Common causes of ICUAW include critical illness polyneuropathy (CIP) and myopathy (CIM), which are revealed by appropriate nerve conduction studies and electromyography.^(1,7)^ Because these electrophysiological studies are time-consuming and require specialized personnel, simplified tests have been proposed to be used as screening tests.^(8)^ Unilateral peroneal and sural nerve conduction studies can accurately screen for CIP and CIM in ICU patients.^(9)^ A single nerve test (the peroneal nerve test) has been validated in two multicenter studies as a 100% sensitivity test compared to a complete nerve conduction study and electromyography in the diagnosis of CIP/CIM,^(10)^ and it can be performed in 10 minutes.^(11)^ A reduced amplitude of the muscle action potential obtained after direct muscle stimulation can identify muscle membrane excitability and CIM in non-cooperative patients and can be useful in differentiating CIM from CIP in the ICU. Prolonged duration of the compound muscle action potential amplitude, which is obtained during a conventional nerve conduction study, can also suggest CIM (Figure 1).^(1)^ Differential diagnosis between CIP and CIM is important because prognosis can be better for CIM than for CIP.^(12,13)^

ICUAW is a clinically relevant complication during the acute stage of disease and after discharge from the acute-care hospital. In the ICU, severe muscle weakness is independently associated with prolonged mechanical ventilation, ICU stay, hospital stay and increased mortality.^(1)^ Patients developing weakness during the ICU stay have reduced quality of life and increased mortality 1 year after ICU discharge.^(14)^ In survivors of acute lung injury, ICUAW resolves within several weeks to months in most patients, but it can persist longer in other patients.^(15,16)^ In a recent Brazilian cohort study, physical activity, muscle strength and exercise capacity were significantly reduced in survivors of severe sepsis and septic shock.^(17)^ Physical dysfunction, either measured using objective physical function tests, such as the 6-minute walking distance test, or subjectively perceived by the patients as weakness, persists longer than muscle weakness and can be a major problem affecting the quality of life even in patients who regain their full muscle strength. There can be several reasons for this, not least that the outcome is affected by a myriad of factors.^(18)^

In conclusion, muscle weakness acquired during the ICU stay is a clinically relevant complication with an impact on early and late outcome. Timely diagnosis is much deserved for patients, and pragmatic diagnostic flow-charts, as proposed here, may be of help in daily practice.

## References

[1] Latronico N, Bolton CF (2011). Critical illness polyneuropathy and myopathy: a major cause of muscle
weakness and paralysis. Lancet Neurol.

[2] Argov Z, Latronico N (2014). Neuromuscular complications in intensive care patients. Handb Clin Neurol.

[3] Hermans G, Clerckx B, Vanhullebusch T, Segers J, Vanpee G, Robbeets C (2012). Interobserver agreement of Medical Research Council sum-score and
handgrip strength in the intensive care unit. Muscle Nerve.

[4] Vanhoutte EK, Faber CG, van Nes SI, Jacobs BC, van Doorn PA, van Koningsveld R, Cornblath DR, van der Kooi AJ, Cats EA, van den Berg LH, Notermans NC, van der Pol WL, Hermans MC, van der Beek NA, Gorson KC, Eurelings M, Engelsman J, Boot H, Meijer RJ, Lauria G, Tennant A, Merkies IS, PeriNomS Study Group (2012). Modifying the Medical Research Council grading system through Rasch
analyses. Brain.

[5] Parry SM, Berney S, Granger CL, Dunlop DL, Murphy L, El-Ansary D (2015). A new two-tier strength assessment approach to the diagnosis of
weakness in intensive care: an observational study. Crit Care.

[6] Vanpee G, Hermans G, Segers J, Gosselink R (2014). Assessment of limb muscle strength in critically ill patients: a
systematic review. Crit Care Med.

[7] Friedrich O, Reid MB, Van den Berghe G, Vanhorebeek I, Hermans G, Rich MM (2015). The Sick and the Weak: Neuropathies/Myopathies in the Critically
Ill. Physiol Rev.

[8] Latronico N, Smith M. (2014). Introducing simplified electrophysiological test of peripheral nerves
and muscles in the ICU: choosing wisely. Intensive Care Med.

[9] Moss M, Yang M, Macht M, Sottile P, Gray L, McNulty M (2014). Screening for critical illness polyneuromyopathy with single nerve
conduction studies. Intensive Care Med.

[10] Latronico N, Bertolini G, Guarneri B, Botteri M, Peli E, Andreoletti S (2007). Simplified electrophysiological evaluation of peripheral nerves in
critically ill patients: the Italian multi-centre CRIMYNE study. Crit Care.

[11] Latronico N, Nattino G, Guarneri B, Fagoni N, Amantini A, Bertolini G, GiVITI Study Investigators (2014). Validation of the peroneal nerve test to diagnose critical illness
polyneuropathy and myopathy in the intensive care unit: the multicentre Italian
CRIMYNE-2 diagnostic accuracy study. F1000Res.

[12] Guarneri B, Bertolini G, Latronico N (2008). Long-term outcome in patients with critical illness myopathy or
neuropathy: the Italian multicentre CRIMYNE study. J Neurol Neurosurg Psychiatry.

[13] Koch S, Wollersheim T, Bierbrauer J, Haas K, Mörgeli R, Deja M (2014). Long-term recovery In critical illness myopathy is complete, contrary
to polyneuropathy. Muscle Nerve.

[14] Hermans G, Van Mechelen H, Clerckx B, Vanhullebusch T, Mesotten D, Wilmer A (2014). Acute outcomes and 1-year mortality of intensive care unit-acquired
weakness. A cohort study and propensity-matched analysis. Am J Respir Crit Care Med.

[15] Fan E, Dowdy DW, Colantuoni E, Mendez-Tellez PA, Sevransky JE, Shanholtz C (2014). Physical complications in acute lung injury survivors: a two-year
longitudinal prospective study. Crit Care Med.

[16] Needham DM, Wozniak AW, Hough CL, Morris PE, Dinglas VD, Jackson JC, Mendez-Tellez PA, Shanholtz C, Ely EW, Colantuoni E, Hopkins RO, National Institutes of Health NHLBI ARDS Network (2014). Risk factors for physical impairment after acute lung injury in a
national, multicenter study. Am J Respir Crit Care Med.

[17] Borges RC, Carvalho CR, Colombo AS, da Silva Borges MP, Soriano FG (2015). Physical activity, muscle strength, and exercise capacity 3 months
after severe sepsis and septic shock. Intensive Care Med.

[18] Latronico N, Herridge MS (2015). Unraveling the myriad contributors to persistent diminished exercise
capacity after critical illness. Intensive Care Med.

